# Functional capacity in sickle cell disease: A pilot study with 1-minute sit-to-stand test

**DOI:** 10.1016/j.htct.2025.106230

**Published:** 2026-01-10

**Authors:** Michele Barroso Thomaz, Lucas Fernandes Suassuna, Júlia Campos Fabri, Isabela de Oliveira Araújo, Júlia Carneiro Almeida, Daniela de Oliveira Werneck Rodrigues

**Affiliations:** a Fundação Hemominas, Juiz de Fora, MG, Brazil; b Universidade Federal de Juiz de Fora (UFJF), Juiz de Fora, MG, Brazil

**Keywords:** Functional status, Sickle cell disease, Quality of life, 1-minute sit-to-stand test

## Abstract

**Background:**

Sickle cell disease, the most prevalent monogenic recessive genetic disorder in the world, is characterized by two main pathogenic mechanisms: vaso-occlusion and hemolysis. These characteristics lead to reduced tolerance to physical exertion and, consequently, a reduced functional capacity which can be assessed using the one-minute sit-to-stand test. Complications from sickle cell disease result in poor quality of life, increased absenteeism from school and work, and impaired social interaction.

**Method:**

Between January 2023 and April 2024, a pilot cross-sectional study was conducted with sickle cell disease patients aged from 18 to 60 years. The one-minute sit-to-stand test, Borg's perceived exertion scale, and the SF-36 quality of life questionnaire were utilized. Patients were monitored during the test. The sample was dichotomized based on test performance and SF-36 scores. Furthermore, clinical and demographic variables were analyzed.

**Main results:**

Fifty-eight individuals participated in the final analysis. The mean age was 29.84 ± 11.20 years; 55.1 % were men, and 79.3 % identified themselves as Black or mixed race. The most prevalent genotype was hemoglobin SS (67.2 %), and 77.5 % were taking Hydroxyurea. The group with a better performance in the one-minute sit-to-stand test showed better quality of life as assessed using the SF-36 questionnaire.

**Conclusion:**

Functional capacity is a significant factor in the autonomy and quality of life of patients with sickle cell disease. The one-minute sit-to-stand test is a low-cost and easily applicable test, which can contribute to the assessment of functional capacity in the routine follow-up of these patients.

## Introduction

Sickle cell disease (SCD), resulting from a mutation in the hemoglobin (Hb) gene that leads to the formation of Hb S, is the most prevalent recessive monogenic disease in the world. There are multiple genotypes that cause SCD including the severe homozygous form, Hb SS, or as heterozygous forms with other hemoglobin variants, such as Hb C or Hb β-thalassemia [1].

When deoxygenated, Hb S polymerizes and modifies the morphology of red blood cells making their membrane rigid and into a sickle shape. This modified state favors the occurrence of vaso-occlusive crises (VOC), endothelial dysfunction, hemolysis, and activation of inflammatory responses through the release of cytokines and reactive oxygen species. VOC lead to ischemia and are the main factor responsible for pain crises in SCD as well as other complications, such as acute chest syndrome and osteonecrosis. Endothelial dysfunction is associated with intravascular hemolysis, nitric oxide depletion and hypercoagulability, and is the underlying mechanism of complications such as strokes, pulmonary hypertension and organ damage, especially of the kidneys and heart [1,2].

The complex physiopathology of SCD reduces tolerance to physical exertion, which is seen by a reduced functional capacity (FC). The acute complications of SCD result in school and work absenteeism, negative impacts on socialization and a low quality of life. Therapeutic exercise is an important tool for rehabilitation in SCD, recovering cardiorespiratory capacity and autonomy, and improving socialization. However, the prescription of exercise in SCD is a highly complex topic and may cause VOC if excessively intense [2].

Assessing FC is crucial for developing an appropriate exercise prescription, as a wide array of functional assessment tools, ranging from simple field tests to sophisticated laboratory procedures, is available. The cardiopulmonary exercise test (CPET) is the gold standard; however, it requires specific, costly equipment that limits its availability [3]. Other methods for functional assessment consist in field tests, which include the ergometric test, Bruce protocol, 6-Minute Walk Test (6MWT), and the 1-Minute Sit-to-Stand test (1-MSTST) [4,5]. The 1-MSTST is a submaximal test validated for assessing FC, postural control, proprioception, and lower limb strength. It is a well-tolerated, low-cost test that can be applied domestically, at the bedside and in small rooms as it only requires an armless chair, a stopwatch and minimal floor space [6,7]. To the present moment, there are no studies assessing FC in SCD patients utilizing the 1-MSTST.

## Objective

The objective of this study is to assess FC in adult SCD patients using the 1-MSTST test, and to evaluate possible associations between the FC, quality of life, and clinical and demographic variables.

## Method

This cross-sectional pilot study to assess FC in SCD patients using the 1-MSTST was carried out between January and April 2024. Participants were active patients in a secondary care facility specialized in hematology and hemotherapy. Adults aged between 18 and 60 years, diagnosed with SCD, who signed an informed consent form, participated in the study. Exclusion criteria were: refusal to participate; clinical events during the 1-MSTST; a drop in oxygen saturation >4 % during the 1-MSTST; systolic blood pressure >180 mmHg or diastolic blood pressure >110 mmHg; unstable angina; resting heart rate >120 bpm; and orthopedic conditions that limited performance in the 1-MSTST.

The instruments utilized for this study were: the 1-MSTST test, the Borg rate of perceived exertion scale for the assessment of work of breathing, [8] and the Short Form 36 Health Survey Questionnaire, [9] which is divided in eight domains (Physical functioning; physical role limitations; bodily pain; general health perceptions; energy/vitality; social functioning; emotional role limitations; and mental health) and two summary measures (physical component summary and mental component summary). All anthropometric and clinical data were collected with standardized equipment and all tests were performed supervised by trained research staff. A brochure containing information about lifestyle changes and possible physical activities was created by the research staff and handed to the study participants.

Initially, a descriptive analysis was performed utilizing the adequate frequency and central tendency measures. The study by Strassman et al. was utilized to obtain the age-corrected target repetitions for the 1-MSTST [10] by which our sample was standardized. For the comparative analysis, the sample was divided in two groups based on the median 1-MSTST performance, and analyzed with Student’s *t*-test and Pearson’s chi-squared test. The variables included in the analysis were age, gender, ethnicity, SCD genotype, use of hydroxyurea, 1-MSTST performance, baseline and post-intervention heart rate, baseline and post-intervention peripheral oxygen saturation, and baseline and post-intervention Borg scale. The software IBM SPSS Statistics 25® was utilized for the statistical analysis. This study was registered and approved by the institution’s research ethics committee under the number CAAE 63,424,422.9.0000.5118.

## Results

**Figure 1:**
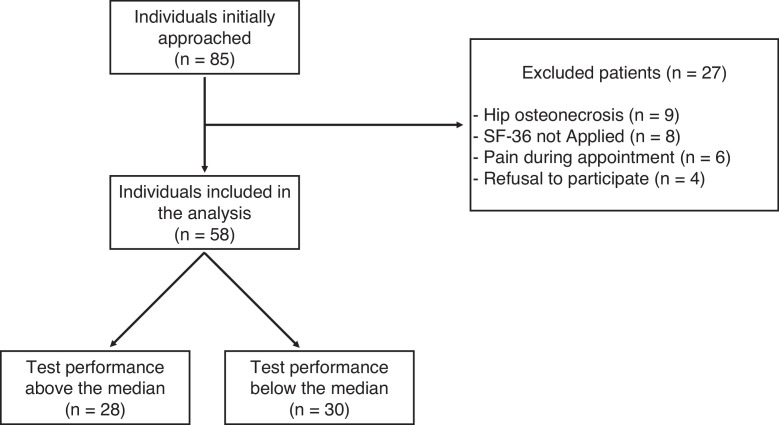
Study flowchart.

In total, 85 individuals met the inclusion criteria of the study; however, 27 were later excluded, as nine had a diagnosis of hip osteonecrosis, eight did not complete the SF-36 health survey questionnaire, six presented with lower body pain during the clinical assessment and four refused to participate. After the 58 participants performed the 1-MSTST, the sample was divided in two groups based on the median performance (20.75 repetitions): 28 patients had an above median performance and 30 patients had a below median performance (Figure 1).

Of the 58 participants, the average age was 29.84 ± 11.20 years with ages ranging between 18 and 59 years, 55.1 % were male, and 79.3 % identified themselves as Black. The most frequent genotype was Hb SS (*n* = 39; 67.2 %) and 77.5 % of the sample was taking hydroxyurea. In the comparative analysis, the group with the best 1-MSTST performance had a statistical tendency to be younger (p-value = 0.08), and had a higher post-intervention heart rate (p-value = 0.001). The other analyzed variables were not statistically significant, such as use of hydroxyurea and disease genotype (Table 1).

**Table 1:** Demographic and clinical variables of the sample categorized by 1-MSTST performance.

Variable	Total	Median performance		p-value
		<20.75 repetitions (*n* = 30)	>20.75 repetitions (*n* = 28)	
Age (mean)	29.84 ± 11.20	32.27 ± 11.95	27.25 ± 9.89	0.08
Gender, male – n (%)	32 (55.1)	15 (50)	17 (60.7)	0.41
Race, black – n (%)	46 (79.3)	24 (80)	22 (78.6)	0.57
Genotype – n (%)				0.75
Hb SS	39 (67.2)	20 (66.7)	19 (67.9)	
Hb Sβ^+^	9 (15.5)	5 (16.7)	4 (14.3)	
Hb SC	9 (15.5)	4 (13.3)	5 (17.9)	
Hb Sβ^0^	1 (1.7)	1 (3.3)	0 (0)	
Medication				
Hydroxyurea use	45 (77.5 %)	23 (76.7 %)	22 (78.6 %)	0.86
Average dosage (mg)	1130.16 ± 360.57	1133.70 ± 400.36	1126.45 ± 323.19	0.94
1-MSTST				
Repetitions	20.44 ± 5.88	15.96 ± 3.86	25.25 ± 3.29	<0.001*
Target/real performance ratio (%)	43.86 ± 11.79	34.94 ± 7.51	53.42 ± 7.07	<0.001*
Baseline heart rate (bpm)	78.69 ± 13.10	76.66 ± 11.28	80.87 ± 14.70	0.22
Post intervention heart rate (bpm)	121.05 ± 20.42	112.73 ± 20.84	129.96 ± 15.95	0.001*
Baseline O_2_ saturation (%)	93.68 ± 4.01	93.43 ± 4.57	93.96 ± 3.36	0.61
Post intervention O_2_ saturation (%)	92.81 ± 4.39	92.70 ± 5.22	92.94 ± 3.37	0.83
Baseline Borg	1.61 (0 - 2.12)	1.85	1.35	0.36
Post intervention Borg	5 ± 2.13	5.1 ± 2.37	4.89 ± 1.88	0.71

1-MSTST: 1-minute sit-to-stand test.

The data regarding the results of the SF-36 health survey analysis are found in Table 2. The group with a worse performance in the 1-MSTST had statistically significant lower scores in the physical component summary (p-value = 0.05), mental health (p-value = 0.01) and general health perceptions (p-value = 0.02). Other SF-36 health survey dimensions, such as physical role limitations (p-value = 0.07) and physical functioning (p-value = 0.09) approached statistical significance for a worse score in the group with the worst performance.

**Table 2:** Results of SF-36 health survey analysis categorized by 1-MSTST performance.

SF-36	Total	Median performance		p-value
		<20.75 repetitions (*n* = 30)	>20.75 repetitions (*n* = 28)	
PCS	44.45 ± 9.74	42.12 ± 9.27	46.95 ± 9.76	0.05
MCS	47.97 ± 12.38	45.39 ± 13.23	50.73 ± 10.96	0.10
Physical functioning	72.98 ± 20.82	68.16 ± 20.11	77.50 ± 21.01	0.09
Physical role limitations	60.52 ± 44.30	52.50 ± 45.65	73.21 ± 39.63	0.07
Emotional role limitations	68.41 ± 38.03	60.00 ± 40.49	73.80 ± 37.79	0.18
Energy/vitality	59.91 ± 28.30	54.33 ± 25.68	63.39 ± 29.44	0.21
Mental health	68.70 ± 25.51	61.86 ± 25.70	76.71 ± 20.07	0.01*
Social functioning	79.60 ± 29.37	76.66 ± 30.21	83.48 ± 28.06	0.37
Bodily pain	76.57 ± 30.23	66.30 ± 33.52	78.78 ± 29.30	0.13
General health perceptions	50.70 ± 25.16	44.86 ± 20.53	59.32 ± 26.27	0.02*

SF-36: SF-36 health survey questionnaire; PCS: Physical component summary; MCS: Mental component summary.

## Discussion

SCD is one the most epidemiologically relevant hematological conditions in the world. In Brazil it represents an important health burden, affecting an estimated 60,000–100,000 people [11]. Between 2014 and 2020, an annual average of 1087 people were born with the disease in Brazil, with an incidence of 3.75 cases per 10,000 newborns [12]. SCD represents a challenge for the Brazilian public health system due to its complex pathophysiology, need for interdisciplinary care, and socioeconomic impact. Historically, newborn screening and continued health education, in addition to regular medical care, have been effective strategies for reducing complications and increasing quality of life for SCD patients in Brazil [1].

Patients were not included in the analysis of this study if they presented with conditions that could interfere with their performance in the 1-MSTST, the most prevalent of which was hip osteonecrosis, with a frequency of 10.58 %. Osteonecrosis is a frequent complication of SCD that occurs due to VOC with the most frequent sites being the hips, vertebrae and shoulders [13]. The prevalence of osteoarticular involvement increases with age. Ouederni et al. found a prevalence of hip osteonecrosis of 18.3 % in 20-year-old SCD patients, compared to a 2.3 % hip osteonecrosis prevalence in 10-year-olds [14,15]. Daltro et al. reported a prevalence of osteonecrosis in 11.1 % of SCD patients in Brazil, most frequently of the hip joint (74.6 %), [15] a result similar to the current findings.

Regarding the SCD genotype, 67.1 % of the present sample had the Hb SS genotype, a proportion similar to other Brazilian studies, such as Silva et al., whose group identified a slightly greater prevalence of Hb SS, at 75 % [16]. Souza et al. identified a lower prevalence of 60 % in their sample with Hb SS [17]. These variations are common and reflect differences in recruiting methods and populations. Cardoso et al. found a median age of 28.6 ± 9.9 years and a 65 % prevalence of Hb SS, very similar results to this study [18]. These findings reinforce the representativeness of the current sample. The different genotypes of SCD correlate to disease severity and symptomatology: Hb Sβ^+^-thalassemia and Hb SC are considered of mild severity, while Hb SS and Hb Sβ^0^-thalassemia present a greater clinical relevance and severity [19]. In this study, the SCD genotype was not significantly associated with worse 1-MSTST performance, possibly due to the high proportion of Hb SS in the sample (*n* = 39; 67.2 %).

A worse performance in the 1-MSTST was observed among older participants (p-value = 0.08), which may be explained by the buildup of osteoarticular and inflammatory damage caused by SCD and aging. Other sociodemographic variables, such as gender and ethnicity, were not statistically significant in this study. Hydroxyurea is the most efficient pharmacological therapy for SCD available in Brazil; its use is associated with a lower rate of degenerative damage [19,20]. In the present study, 77.5 % of the participants were in continuous use of hydroxyurea; however, its use was not associated with a better performance in the 1-MSTST (p-value = 0.86). The authors did not find any literature regarding 1-MSTST performance and use of hydroxyurea, or other drugs, among SCD patients.

No participants of this study were capable of reaching their age-corrected performance target for the 1-MSTST. Studies utilizing other methods to assess FC in SCD patients present with similar results. Cardoso et al. assessing the FC in an adult population with sickle cell anemia utilizing the 6MWT found that the average distance covered by study participants was less than the expected target distance for the Brazilian population (335.3 ± 70.6 m versus 504.7 ± 5.5 m; p-value <0.001) [21]. The 1-MSTST is a low-cost, simple assessment that can be applied in domestic and bedside environments; it provides a similar hemodynamic and ventilatory response to the 6MWT, which is considered the gold-standard field FC assessment tool. There are no studies utilizing the 1-MSTST to assess FC in SCD, which is a limiting factor of the present discussion.

The association between quality of life and FC is evident in several health conditions, including SCD, in which individuals present with decreased tolerance to physical exertion, resulting in loss of muscle mass and adverse psychosocial effects. In addition, it is common for SCD patients to be advised to avoid physical activities, leading to a sedentary lifestyle and increasing adverse psychosocial and body composition effects [22, 23, 24]. A study with SCD patients implemented an eight-week exercise program, with results showing increases in both the FC and quality of life assessed by the SF-36 health survey [23]. The present study found that a worse performance in the 1-MSTST was associated with a worse quality of life assessed by the SF-36 health survey questionnaire. Therefore, in patients with physical disabilities caused by SCD, an approach focused on mental health, in addition to physical training, is of utmost importance, as anxiety and depressive symptoms are very common in this population [24].

## Conclusion

In this study, a better performance in the 1-MSTST was associated with a better quality of life assessed using the SF-36 health survey, however there was no statistical association with any of the clinical and demographic variables. The 1-MSTST, a low cost and easily applicable tool for FC assessment, could be a useful addition to the physical evaluation of SCD patients. The multidisciplinary team could use this test for triage during routine follow-ups and stimulate early physical health interventions for the purpose of reducing morbidity associated with SCD.

## Conflicts of interest

None.
